# Skin Aging and the Upcoming Role of Ferroptosis in Geroscience

**DOI:** 10.3390/ijms25158238

**Published:** 2024-07-28

**Authors:** Rita Rezzani, Gaia Favero, Giorgia Cominelli, Daniela Pinto, Fabio Rinaldi

**Affiliations:** 1 Anatomy and Physiopathology Division, Department of Clinical and Experimental Sciences, University of Brescia, 25123 Brescia, Italy; gaia.favero@unibs.it (G.F.); giorgia.cominelli@unibs.it (G.C.); 2 Interdipartimental University Center of Research “Adaption and Regeneration of Tissues and Organs (ARTO)”, University of Brescia, 25123 Brescia, Italy; dpinto@giulianipharma.com (D.P.); fabio.rinaldi@studiorinaldi.com (F.R.); 3 Italian Society for the Study of Orofacial Pain (Società Italiana Studio Dolore Orofacciale—SISDO), 25123 Brescia, Italy; 4 Human Microbiome Advanced Project Institute, 20129 Milan, Italy

**Keywords:** aging, autoimmune diseases, cutaneous diseases, ferroptosis, gut microbiota, melanoma, skin

## Abstract

The skin is considered the most important organ system in mammals, and as the population ages, it is important to consider skin aging and anti-aging therapeutic strategies. Exposure of the skin to various insults induces significant changes throughout our lives, differentiating the skin of a young adult from that of an older adult. These changes are caused by a combination of intrinsic and extrinsic aging. We report the interactions between skin aging and its metabolism, showing that the network is due to several factors. For example, iron is an important nutrient for humans, but its level increases with aging, inducing deleterious effects on cellular functions. Recently, it was discovered that ferroptosis, or iron-dependent cell death, is linked to aging and skin diseases. The pursuit of new molecular targets for ferroptosis has recently attracted attention. Prevention of ferroptosis is an effective therapeutic strategy for the treatment of diseases, especially in old age. However, the pathological and biological mechanisms underlying ferroptosis are still not fully understood, especially in skin diseases such as melanoma and autoimmune diseases. Only a few basic studies on regulated cell death exist, and the challenge is to turn the studies into clinical applications.

## 1. Introduction

The skin covers the entire outside of the body [1,2,3], and it is considered the most important organ system in mammals [4,5]. Today, with the increasing aging population, it is important to define the metabolic pathways that induce skin aging to evaluate anti-aging therapeutic strategies [5].

**Figure 1:**
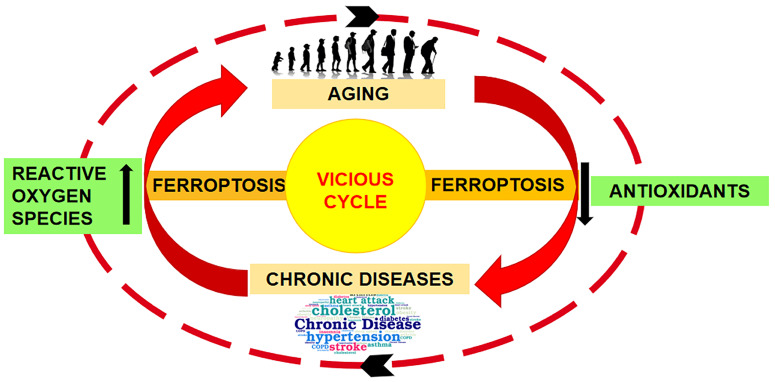
Aging and ferroptosis. The illustration represents the aging induction of ferroptosis, which, in turn, disrupts the imbalance between oxidative stress and antioxidant defense, thereby implementing, in a vicious cycle, the aging-related damage. Illustration from Mazhar et al., 2021 [7]. (This is an open access article distributed under the terms of the Creative Commons Attribution 4.0 International license).

To this purpose, it needs to identify the types of damages that are involved in initiating, maintaining, and sustaining the aging mechanisms [6,7]. The relationship between aging and chronic diseases is represented by the excessive production of reactive oxygen species (ROS), lipid accumulation, redox imbalances, inflammation, and decreased antioxidant systems throughout the body. All these factors lead to conventional regulated cell death (RCD) such as necrosis, apoptosis, and pyroptosis; currently a new mechanism on iron-dependent cell death, ferroptosis is of particular interest for its involvement in the etiopathogenesis of various diseases, including cutaneous pathologies [7,8,9] (Figure 1). 

**Figure 2:**
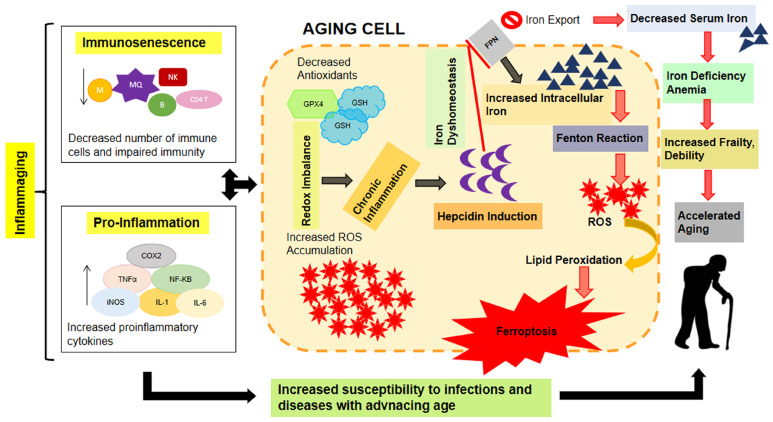
Aging, iron dyshomeostasis, ferroptosis, and hepidicin. The illustration represents a possible interaction among aging, iron dyshomeostasis, ferroptosis, and hepidicin, a hepatic iron-regulatory hormone. Aging increases iron stores in tissues, and the intracellular iron induces redox imbalances and cellular injury, leading to ferroptosis, which, in turn, promotes aging and associated morbidity. The aging-related increment in intracellular iron levels may be linked to the increased production of hepcidin due to underlying chronic inflammation. (GPX4): glutathione peroxidase-4; (GSH): glutathione; (NK): natural killer; (B): B lymphocytes; (CD4 T): CD4 T lymphocyte; (M): microfold cells; (MQ): macrophages; (COX2): cyclooxygenase-2; (TNF-α): tumor necrosis factor alpha; (NF-kB): nuclear factor kappa-light-chain-enhancer of activated B cells; (iNOS): inducible nitric oxide synthase; (IL-1): interleukin-1; (IL-6): interleukin-6; (ROS): reactive oxygen species; (FPN): ferroportin. Illustration from Mazhar et al., 2021 [7]. (This is an open access article distributed under the terms of the Creative Commons Attribution 4.0 International license).

It is known that iron is an important nutrient for humans, but its level increases with aging, and this induces deleterious effects on cellular functions, contributing to morbidity and increased mortality [9,10,11]. Therefore, evaluating aging mechanisms, such as inflammation and iron dyshomeostasis, could be a possible target to gain benefit in controlling aging and comorbidities [7] (Figure 2).

To our knowledge, ferroptosis is not well evaluated in terms of age-related skin changes and pathology compared to other diseases.

The purpose of this review is to highlight morphological and structural changes related to aging and to discuss recent insights into iron-dependent cell death, termed ferroptosis [12,13,14]. Considering the regulation of the processes in which ferroptosis is involved can provide an important insight into its role during life and diseases, helping to provide and optimize therapeutic treatments.

### 1.1. Normal Skin Morphology

**Figure 3:**
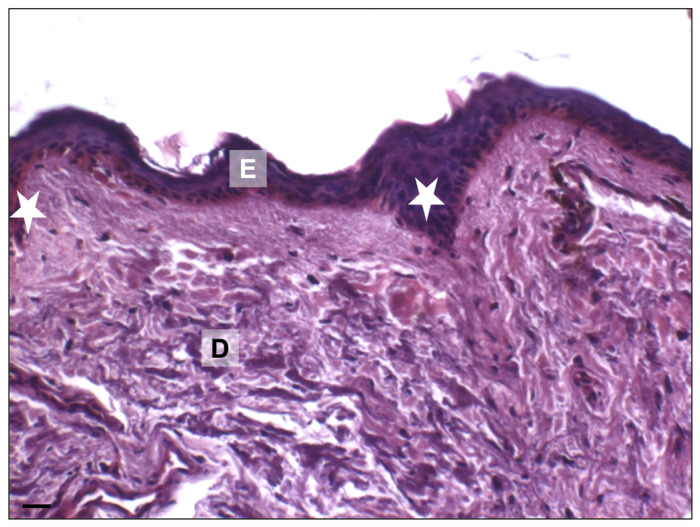
Adult human skin. Representative photomicrograph of face skin from adult donors (under 65 years old). Haematoxylin-eosin staining. The skin biopsies were obtained from head cadaveric specimens (MedCure, Amsterdam, The Netherlands). Specimens were stored at −20 °C, defrosted before the anatomical dissecting session, and analyzed at the Anatomical Facility “Luigi Fabrizio Rodella” of the University of Brescia (Italy). The human cadaveric studies have been performed in accordance with the ethical standards laid down in the 1964 Declaration of Helsinki and its later amendments. Bar: 20 µm. (E): epidermis; (D): dermis; (white stars): epidermal network ridges. Illustration adapted from Favero et al., 2024 [18].

The skin is composed of three layers: epidermis, dermis, and subcutaneous tissue, or hypodermis [15,16,17]. Today, its cytoarchitecture is well known, and Figure 3 shows normal skin layers obtained from adult cadavers (50–60 years old) that are present at the Anatomical Facility “Luigi Fabrizio Rodella” of the University of Brescia (Italy) [18]. The skin has normal epidermal thickness and a well-organized dermis layer. The basal layer in youthful skin is undulated with network ridges, as shown in Figure 3. Network ridges penetrate the dermis, and this network is important for maintaining the integrity of skin morphology, contributing to skin function, and a continuous renewal process [19].

The epidermis is formed of a specific constellation of cells known as keratinocytes, which are the protective barrier against environmental factors and pathogens [5,20]. These cells synthesize keratin, a long, threadlike protein showing a protective function. The epidermis harbors several cell populations, such as melanocytes, Langerhans, Merkel, immune cells, glands, and hair follicles. Melanocytes interact with skin pigmentation, and immune cells act as sentinels, defending the skin against infections and injuries [21]. Dermis is fundamentally made up of fibrillar structural proteins known as collagen and elastin; these proteins are responsible for skin elasticity and firmness [15,22,23]. The hypodermis contains small lobes of fat and cells known as lipocytes [24].

**Figure 4:**
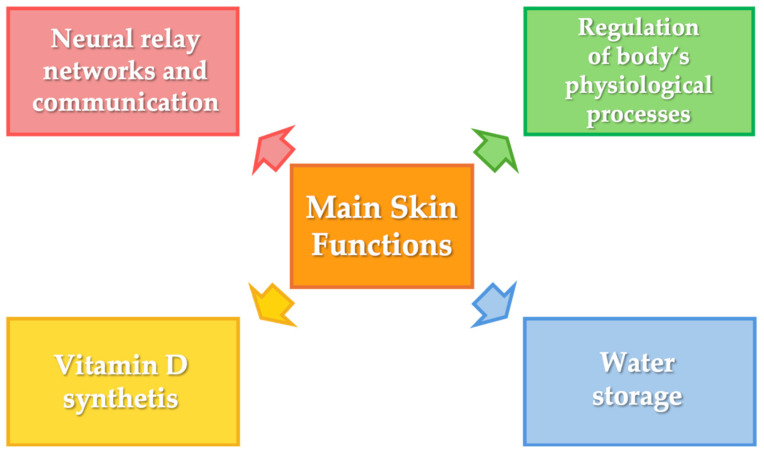
Human skin functions. The illustration summarizes the main human skin functions. Illustration adapted from Ahmed and Mikail et al,. 2024 [3].

All skin cells are strategically organized to execute their role within an intricate skin framework [17]. The framework regulates several physiological processes, including mechanical and environmental protection, thermoregulation, neural relay networks, water storage, and vitamin synthesis (Figure 4).

**Figure 5:**
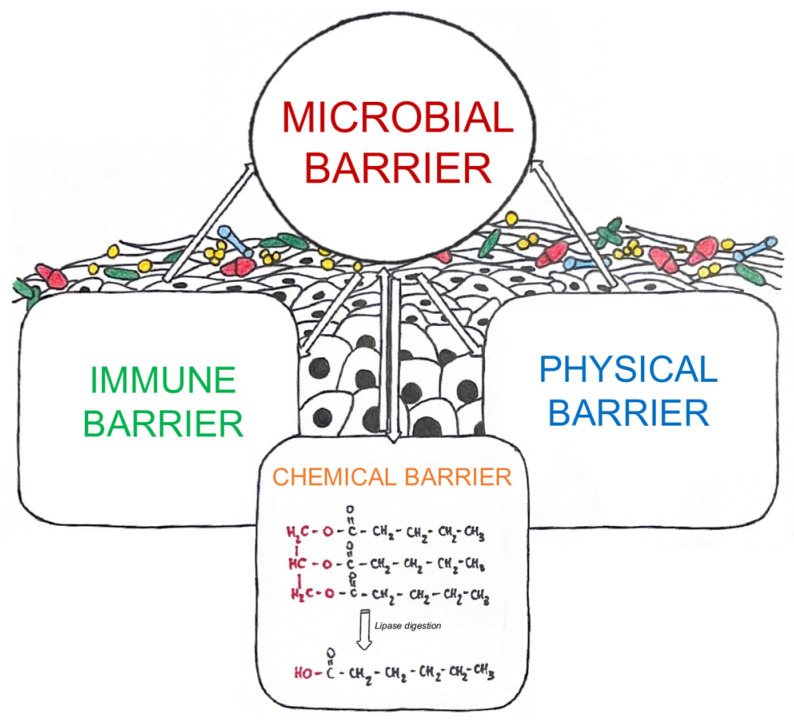
Human skin microbiota. The skin microbiota affects the cutaneous physical barrier function. The microbiota enhances the skin’s chemical barrier by producing lipases that digest sebum triglycerides into free fatty acids, which in turn amplify skin acidity and limit colonization by transient and pathogenic species. Moreover, the skin microbiota stimulates innate and adaptive immune defenses. Illustration adapted from Harris-Tryon and Grice, 2022 [28].

Recently, a strict link between skin and microbiota has been underlined in various tissues and organs, such as the liver, intestines, and kidney, contributing to disease progression. Several studies have shown that millions of different microbes, such as bacteria, viruses, and fungi, inhabit the surface of the human body and create an important health ecosystem. Furthermore, microbes have an essential role in protecting against invading pathogens and in educating our immune system [3,25,26]. The skin microbiota affects all aspects of the skin barrier and interacts directly with commensal and pathogenic microbes encountered on the surface [27,28] (Figure 5).

An altered skin microbiota is more often the rule than the exception in skin diseases. Changes in the availability of resources and, in some cases, the complete devastation of their habitat are factors that result in the depletion of normal skin residents in favor of opportunists. Whether this is a cause or a consequence is unknown, but altered microbial communities can mediate tissue damage and/or inflammation in several skin diseases [28].

It is known that skin damage can affect other barrier sites, such as the lung and intestine [28]. The relationship between skin and gut depends on the production of hyaluronan fragments present in the dermis during injury that stimulate the differentiation of fibroblasts into proinflammatory adipocytes. These cells induce gut inflammation through the production of several inflammatory mediators [29]. In addition, the gut microbiota can also increase the inflammation process at the skin level, inducing a vicious cycle [30]. Understanding how skin microbial communities interact with the host and each other is critical to targeting this interface for the prevention and treatment of skin disorders.

### 1.2. Effects of Aging

**Figure 6:**
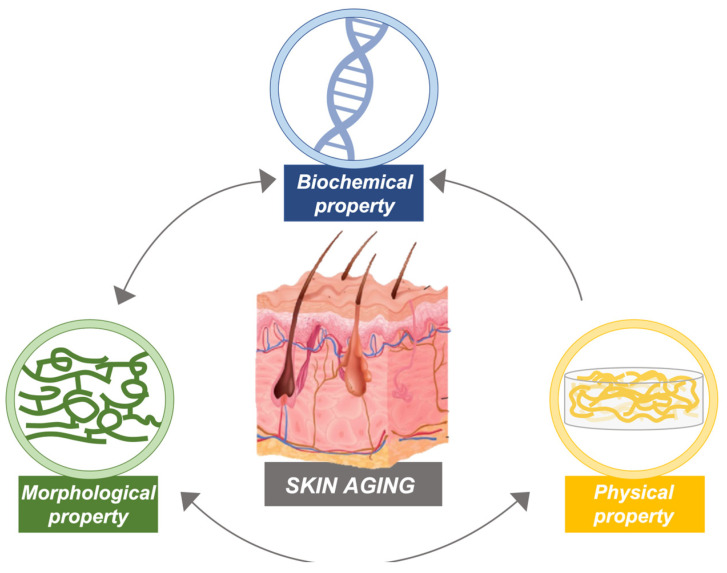
Human skin aging physiopathological process. Human skin aging is related to biomechanical, structural, and physical change at nano-, micro-, and macro-scales. Illustration adapted from Park, 2022 [32].

Aging is the result of a gradual decline at the cellular and organismal levels [31]. Numerous studies have been reported on the effects on several functional properties, such as biochemical, morphological, and physical features, at different scales (nano, micro, and macro), during the aging of the human skin and how such properties are associated [32] (Figure 6).

**Figure 7:**
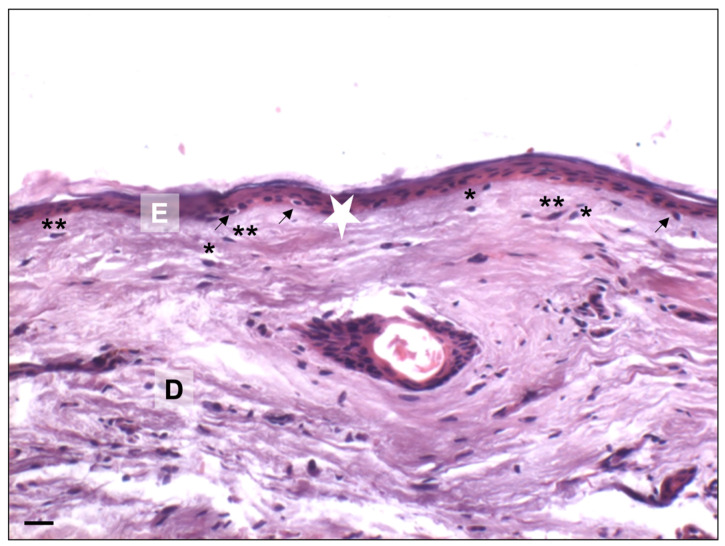
Elderly human skin. Representative photomicrograph of face skin from elderly donors (over 65 years old). Haematoxylin-eosin staining. The skin biopsies were obtained from head cadaveric specimens (MedCure, Amsterdam, The Netherlands). Specimens were stored at −20 °C, defrosted before the anatomical dissecting session, and analyzed at the Anatomical Facility “Luigi Fabrizio Rodella” of the University of Brescia (Italy). The human cadaveric studies have been performed in accordance with the ethical standards laid down in the 1964 Declaration of Helsinki and its later amendments. Bar: 20 µm. (E): epidermis; (D): dermis; (white stars): epidermal network ridges; (arrows): melanocytes; (**): fibroblasts; (*): mast cells. Illustration adapted from Favero et al., 2024 [18].

The well-known skin cytoarchitecture of old people is described in Figure 7. Studied samples have been obtained from the skin of old cadavers (80–90 years old) that are present at the Anatomical Facility “Luigi Fabrizio Rodella” of the University of Brescia (Italy) [18]. Histochemical analyses show a reduction in epidermal layers and a disorganization of collagen fibers in the dermis [15,17,33]. Moreover, the basal layer is not undulated but flat, and this alteration induces a potential reduction in the surface area available for exchanges between the epidermis and dermis. A reduction in surface area can make the epidermis more susceptible to shear forces, leading to increased fragility with altered barrier functions [34].

**Figure 8:**
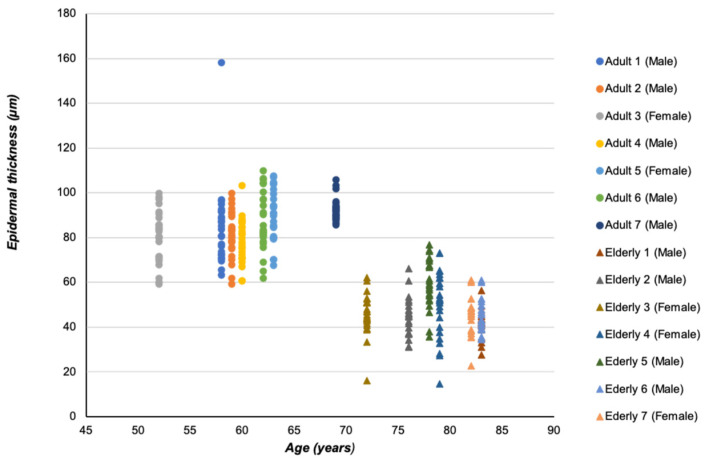
Human epidermal thickness evaluation in adult and elderly specimens. The plot shows the epidermal thickness distribution performed on adult (under 65 years old) and elderly (over 65 years old) donor specimens. The face skin sections were stained with hematoxylin-eosin following standard procedures. The thickness of the epidermal layer of each specimen was calculated in micrometers (μm) by a blind examiner using an image analyzer (Image Pro Premier 9.1; Media Cybernetics, Rockville, MD, USA). The epidermal layer was measured from the free margin of skin to the dermal papillae and epidermal network ridge. The analysis was performed on five alternately stained sections for each skin specimen. Illustration adapted from Favero et al., 2024 [18].

Quantitative analysis of the same adult and elderly donors confirms the reported findings on epidermis thickness, suggesting a significant reduction in the epidermal layer [18] (Figure 8). This may be due to changes in stratum corneum composition, such as reduced levels of natural moisturizing factors and alterations in cell structure [34,35].

**Figure 9:**
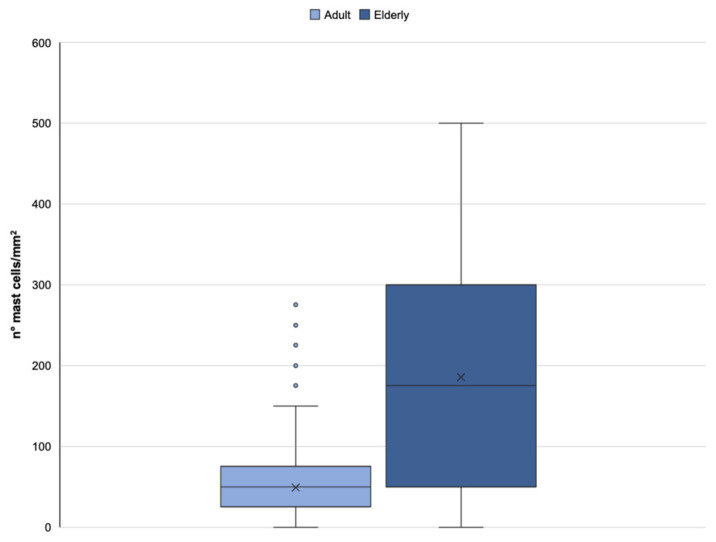
Human dermal mast cell quantification in adult and elderly specimens. The plot summarizes the total number of mast cells in the dermal layer performed on adult (under 65 years old) and elderly (over 65 years old) donor specimens. The face skin sections were stained with toluidine blue. The numbers of mast cells were evaluated by a blind examiner using an optical BX50 microscope (Olympus, Hamburg, Germany) as the number of cells per field. At least five representative visual fields of five alternate sections for each skin specimen were analyzed. Illustration adapted from Favero et al., 2024 [18].

Moreover, Figure 9 shows the number of mast cells in dermis from adult and elderly donors, as reported above [18]. The histochemical results support a significant increase in the number of mast cells in the dermis of elderly donors, confirming that skin aging induces inflammation. The inflammation is partially due to an increase in mast cells. Additionally, it is possible to think that the cytotoxic products of these cells play a role in decreasing the number of fibroblasts in aging skin. This situation shows that mast cells play an important role in skin damage caused by age-related changes. Therefore, mast cells should be considered an important target for anti-aging therapy, as reported by Gunin et al. (2011) [36].

Skin is the site where signs of aging are most visible, and these signs include thin and dry skin, loss of elasticity, and aberrant pigmentation. Intrinsic factors such as hormonal changes and genetic factors are associated with extrinsic factors such as ultraviolet (UV) radiation or pollution; together, these factors determine skin senescence.

**Figure 10:**
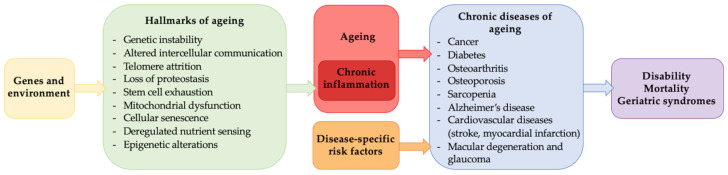
Geroscience and age-related diseases. The illustration represents the link between geroscience and aging. Genetics and environmental factors affect various cellular and physiological pathways fundamental to aging and inflammation. These factors, together with disease-specific risk factors, can increase the risk of aging-related chronic disease development. Illustration adapted from Campisi et al., 2019 [37].

Many molecular and biochemical processes are under investigation in laboratories working on chronic diseases [37]. Interestingly, the relationship among several scientists with different expertise and not only related to lifespan on disease models opened a new field of aging research different from gerontology. The new term used for these studies is “geroscience”, as reported by Kennedy et al. (2014) [38]. Geroscience indicates that aging pathways contribute to many age-related conditions and diseases (Figure 10). The new point of view suggests that multimorbidity is seen as the expression of several systems showing an advanced stage of aging rather than a coincidence of unrelated diseases [39]. Targeting biochemical and molecular pathways of aging ameliorates or prevents multiple clinical problems; this hypothesis remains to be tested by clinical trials, even if it is already supported by several experimental studies [40,41].

## 2. Skin Aging

Skin aging is particularly important for its social impact. The “biological clock” affects the skin and all the organs in a similar way, causing irreversible degeneration [42,43,44]. However, an important dermatologist, Nicholas Perricone, wrote in our book that “wrinkled, sagging skin is not the result of aging. It is a disease, and it is possible to fight it” [45].

Skin aging is induced by both intrinsic and extrinsic factors; the aging processes show phenotypic changes in skin cells and structural/functional alterations in several cellular components that are responsible for elasticity, hydration, and tensile strength [46,47].

### 2.1. Intrinsic Aging

Intrinsic aging depends on time, and this process is usually found in sun-protected areas of the body, i.e., the inner side of arms and legs (Kohl et al., 2011). The aging process in other parts of the body is influenced by extrinsic factors, especially solar UV radiation [48].

**Figure 11:**
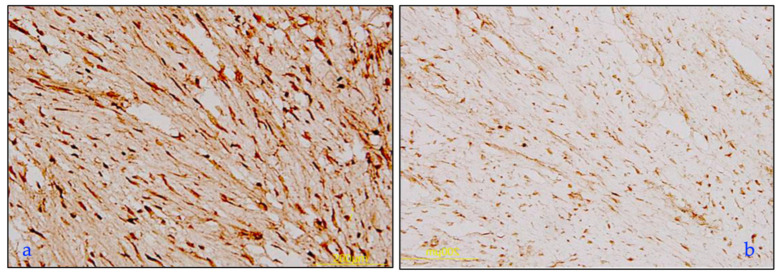
Human skin dermal collagen. Representative photomicrographs of type I (**a**) and type III (**b**) collagen immunohistochemistry at the dermal layer were performed on adult (**a**) and old (**b**) skin specimens. Type I collagen immunostaining was widely distributed, whereas type III collagen immunostaining was light and sparse with an uneven distribution. Bars: 200 µm. Illustration from Cheng et al., 2011 [51]. (This is an open access article distributed under the terms of the Creative Commons CC-BY license).

Intrinsic skin aging determines several histological changes in basal layers of skin with a reduction of cellular proliferation (see Figure 3 and Figure 7). The loss of dermal-epidermal interaction induces a small exchange surface for skin nutrition and fragility [17,49,50]. Furthermore, the dermis became atrophic with a decrease in fibroblasts and a reduction in subcutaneous tissue. It is known that the diameter of collagen fiber decreases with age with an increase in the ratio between type I and III collagen [51,52,53]. The immunohistochemical increase of type I and III collagen, the quantitative analysis, and their ratio from fetus to elderly people are shown in Figure 11 and Table 1, respectively [51].

Therefore, the skewed type I/III ratio leads to alterations in skin tension, elasticity, and healing. Also, the content of type I, III collagen and the type I/III ratio are significantly altered in hypertrophic scar tissue compared to uninjured age-matched controls, resulting in a different structural organization that is mainly determined by patient age [51].

**Figure 12:**
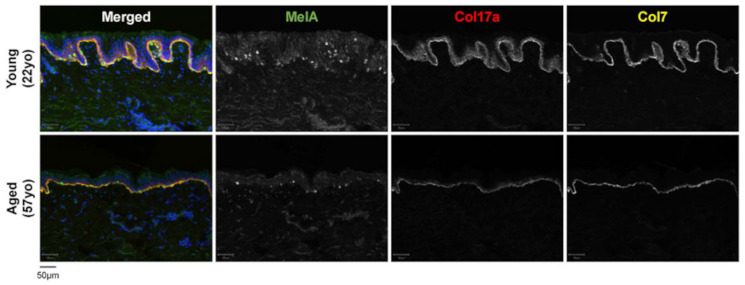
Young and adult sun-protected skin. Representative photomicrographs of skin melanocytes and collagen 17 and collagen 17a immunofluorescence were performed on young (22 years old) and adult (years old) sun-protected skin specimens. Aged skin showed a flattened dermal-epidermal junction, a decreased number of melanocytes, and reduced immunostaining for collagen 17 and 17a. Bars: 50 µm. Illustration from Chin et al., 2023 [31]. (This is an open access article distributed under the terms of the Creative Commons CC-BY license).

Moreover, Chin et al. (2023) showed that collagen 17 and 17a are also involved in the senescence of the skin [31] (Figure 12). During skin aging, the number of melanocytes decreases by approximately 10–20% per decade, and their decrease makes the skin or patches of hypopigmentation appear even if they are in high numbers with respect to keratinocytes (Figure 12).

**Figure 13:**
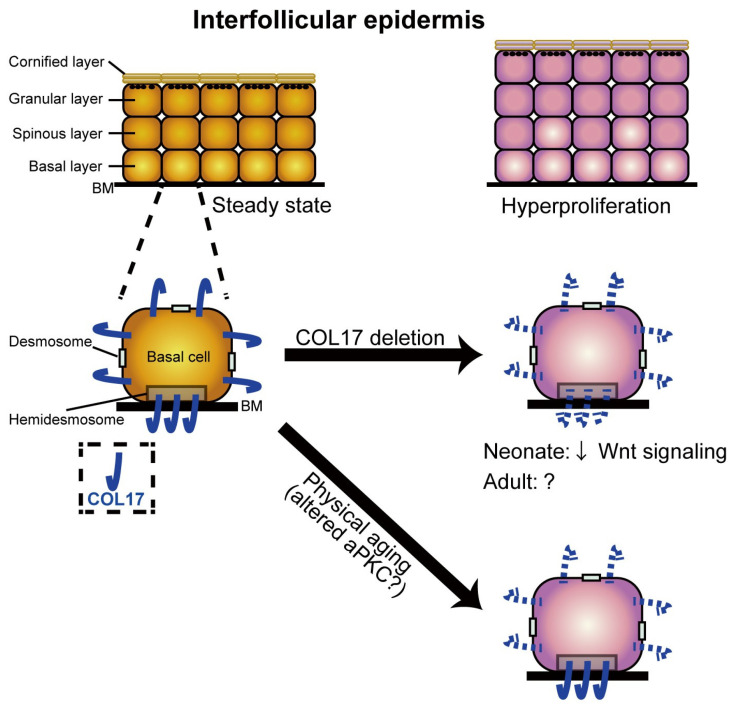
Collagen 17 modulates interfollicular epidermis homeostasis. The illustration represents the possible mechanism of action of collagen 17 in regulating paw interfollicular epidermis homeostasis. Illustration from Watanabe et al., 2017 [54]. (This is an open access article distributed under the terms of the Creative Commons CC-BY license).

Watanabe et al. (2017) identified collagen 17 as a key molecule that controls and modulates epidermal cell division. Additionally, collagen 17 has been shown to form a niche for hair follicle stem cells [54,55,56,57]. However, its role in maintaining the interfollicular epidermis and its stem cells is not fully understood. Watanabe et al. (2017), in an in vivo study, presented the unidentified role of collagen 17 in regulating homeostasis in the paw interfollicular epidermis in terms of proliferation, as summarized in Figure 13 [54]. Collagen 17 deletion in the neonatal epidermis promotes, through the Wnt-β-catenin signaling pathway, the transient hyperproliferation of the interfollicular epidermis. The aging process increased interfollicular epidermis proliferation and promoted the loss of the non-hemidesmosomal collagen 17 distribution associated with altered signaling of a key regulator of epithelial polarity, atypical protein kinase C. Notably, the restoration of collagen 17 in the aged interfollicular epidermis reversed the hyperproliferative skin state. The authors concluded and proposed that collagen 17 may be an attractive transmembrane protein to target and/or prevent epidermal aging and oncogenesis.

Additionally, Wang et al. (2022) observed, through gene knockout and rescue experiments, that collagen 17 may be involved in the formation and stabilization of proper epidermal patterns. Interestingly, aberrant epidermal patterning through collagen 17 deletion is correlated with altered epidermal cell polarity and may involve wound-related skin changes. Collagen 17-negative skin in human junctional epidermolysis bullosa showed a different epidermal pattern with respect to collagen 17-positive skin that resulted from revertant mosaicism. This study emphasized the unrecognized role of collagen 17 in epidermal patterning and underlined that collagen 17 modulation can help prevent epidermal deformation upon wounding [58].

Undoubtedly, collagen 17 is involved in various physiological as well as pathological and physiopathological skin settings, but further studies are needed on this promising topic.

Moreover, blood vessels decrease as well as the autonomic innervation of the epidermis and dermis; skin appendages are affected, and hairs begin to gray with the loss of melanocytes [44,59]. Intrinsic aging has been discussed, and several theories suggest that shortening of telomers, mutations of mitochondrial DNA, a decrease in hormone levels, and oxidative stress are involved in cell aging [60,61].

### 2.2. Extrinsic Aging

Extrinsic aging occurs due to several factors, such as exposure to UV radiation, alcohol intake, over/poor nutrition, and environmental pollution [44]. The exact action spectrum, for example, biological effects depending on wavelengths, remains unclear [62], although both UVA (290–320 nm) and UVB (320–400 nm) are responsible for skin aging [63]. The UV radiations seem to have different actions; UVA radiations induce alterations both in the epidermis and dermis, whereas UVB radiations cause deep damage to the dermis [64]. The function of microvasculature, due to extrinsic aging, declines with aging, as reported above for intrinsic aging. The decline is caused by endothelial dysfunction with impaired vasodilatory function, reduced angiogenic ability, and aberrant expression of adhesion molecules [65,66].

## 3. Features of Aging Cells in the Skin

Aside from morphological cellular alterations, aging cells have several biochemical markers that are identified both in vitro and in vivo [31,67].

An important characteristic of aging cells is the changes in the nuclear envelope, such as the proteins forming the nuclear lamina. Lamin B1 and its receptors decrease during aging, together with the loss of lamin B1 receptors [68]; decrease or loss of lamin is an important marker in many tissues, mainly the skin, for identifying aging cells in response to intrinsic and extrinsic damages [31,69]. The role of the nuclear lamina in remodeling is not well studied, but its function as anchor points for heterochromatin domains that are modified in senescent cells is well known [70]. Alteration of the nuclear lamina induces the expression of many proteins and a change in metabolic profile that is found in aging cells [71]. Aging cells have low mobility and elevated focal adhesion proteins, such as integrins; there is a relationship among integrins, the extracellular matrix, and the cytoskeleton [72]. The integrin proteins can be considered biomarkers, and their regulation can mitigate cellular aging [31]. Furthermore, there are other proteins that have implications for aging cells; some of them are interleukins (ILs) and various metalloproteinases (MMPs) [73]. IL-1β is the most studied senescence-associated secretory phenotype (SASP) protein for its ability to induce paracrine senescence and proliferation arrest, while MMPs are known for their association with damaging effects on senescent dermal fibroblast during tissue damage and for their contribution to skin aging [73,74,75].

## 4. Aging in Cutaneous Compartments and in Skin Cells

As reported above, skin has different compartments and specific types of cells; these cells are crucial in the fields of metabolism and aging. Next, we will report the skin layers in which various types of cells are present to underline their involvement during aging.

### 4.1. Aging in Epidermis

Skin aging shows a higher number of melanocytes than keratinocytes [76,77]. Keratinocytes play an important role in glucose metabolism; with age, the reduced number of these cells and the decrease in their functions determine alterations in glucose uptake, advanced glycation end (AGE) products, and utilization. Reduced glucose uptake impacts energy production, and oxidative stress increases [78]. These mechanisms induce a vicious cycle determining increased levels of oxidative stress, which disrupt glucose metabolism. The results of these processes impair the ability of the cells to counteract the negative effects of intrinsic and extrinsic factors such as free radicals [79]. Then, the epidermis experiences water loss, and the skin is more susceptible to aging processes [80]. Moreover, a normal glucose metabolism is important for providing antioxidant protection and preserving skin health [79,81]. Moreover, glucose is an important source for melanin production; inside the melanocytes, some enzymes use glucose as energy to induce the formation of melanin precursors [82]. An imbalance in glucose levels can lead to alterations in melanin production, contributing to a modification in skin pigmentation [82]. Many proteins are very important for the activities of melanocytes; alterations in their metabolism are responsible for the decreased role of melanocytes, accelerating the formation of age spots and other aging discrepancies [21].

The accumulation of senescent fibroblasts near the dermal-epidermal junctions suggests a relationship between these cells and the hyperpigmentation of senile lentigo [83]. Furthermore, the elimination of senescent fibroblasts using radiofrequency in human volunteers determined the decreased melanin production and pigmentation, as reported by Park et al. (2022) [84]. Other authors suggested that hyperpigmentation is associated, not only with changes in cutaneous aging but also with skin disorders such as melasma [85].

### 4.2. Aging in Dermis

Fibroblasts are the first cells present in the dermis. Several studies indicated that there are many aging fibroblasts in older individuals (>65 years) compared to younger people (<40 years) [86,87]. These cells secrete many MMPs, altering the extracellular matrix with the degradation of collagen and elastin [88]. The process tips the balance towards increased collagen degradation, surpassing the rate of collagen synthesis, and the imbalance induces a gradual loss of skin elasticity with the presence of fine lines and wrinkles [89].

Fibroblast maintains stable cutaneous sugar metabolism, preventing the formation of AGE products; these products are formed when proteins are glycated as a consequence of several amounts of sugar. Then, metabolic changes in the activity of fibroblasts have an important effect on skin aging metabolism [90].

Another useful role of fibroblasts is due to their activity in lipid metabolism; they are defined as the “core” of lipid metabolism. The alteration of lipids alters the integrity of the skin barrier, which induces dehydration and inflammation [5].

### 4.3. Aging in the Subcutaneous Layer

The subcutaneous layer decreases in some areas, especially the face, shins, hands, and feet, while in others, particularly the abdomen in men and the thighs in women, it increases [91]. Changes in the subcutaneous tissue result in changes in the intercellular matrix, a reduction in the number and proliferative and differentiative capacities of preadipocytes, changes in adipocyte size, and changes in adhesion between the tissue and adjacent skin [92]. All these factors modify the mechanical properties of the skin, reducing the cutaneous resistance to mechanical stress and leading to structural instability such as cutaneous wrinkles [93].

The structure of adipocytes and their properties are strongly modified by inflammation, and this finding suggests that inflammation is involved in the alterations of the subcutaneous layer and, therefore, in skin aging. The changes depend quantitatively on the strength of the inflammation because a strong inflammation induces involution of the subcutaneous layer, as reported by Asterholm et coll., (2014) [94]. The inflammation of this layer arises in many cells, including macrophages, and this induces a vicious cycle with the production of other inflammatory cells by preadipocytes rather than adipocytes [95]. This means that preadipocytes have different cytokine production than adipocytes; the cytokines act as chemoattractants for monocytes and macrophages [96]. These cells are responsible for directly binding to Toll-like receptors that stimulate the nuclear factor-kB (NF-kB) signaling pathway and the release of proinflammatory cytokines [97]. These findings have considerable therapeutic relevance because several anti-inflammatory substances have suggested anti-aging activity, as reported by Wollina et al. (2017) [92], (Table 2).

Another important point linked to aging and the subcutaneous layer is the presence of beige and white adipocytes. Beige adipocytes are significantly smaller and contain multilobular lipid droplets and numerous mitochondria that allow them to perform thermogenesis, leading to a reduction in fat mass. Recently, it was demonstrated that the number of beige adipocytes declines with age, and it seems that their transformation from white adipocytes is connected to the development of obesity and other aging disorders [98]. The transformation of white to beige adipocytes can be an antiaging strategy [92]. The mechanical ability of the skin is due to the collagen content present in the subcutaneous layer. Collagen networks, but not adipocytes, mainly determine the mechanical properties of the subcutaneous layer [99]. High fibrous content in the subcutaneous layer has recently been shown to be a negative factor for fat reduction after bariatric surgery [100]. The authors did not find a significant relationship between adipocyte size and fibrotic content, but this finding could be another important hypothesis to consider in clinical trials [100].

### 4.4. Aging in the Immune System Cells

**Figure 14:**
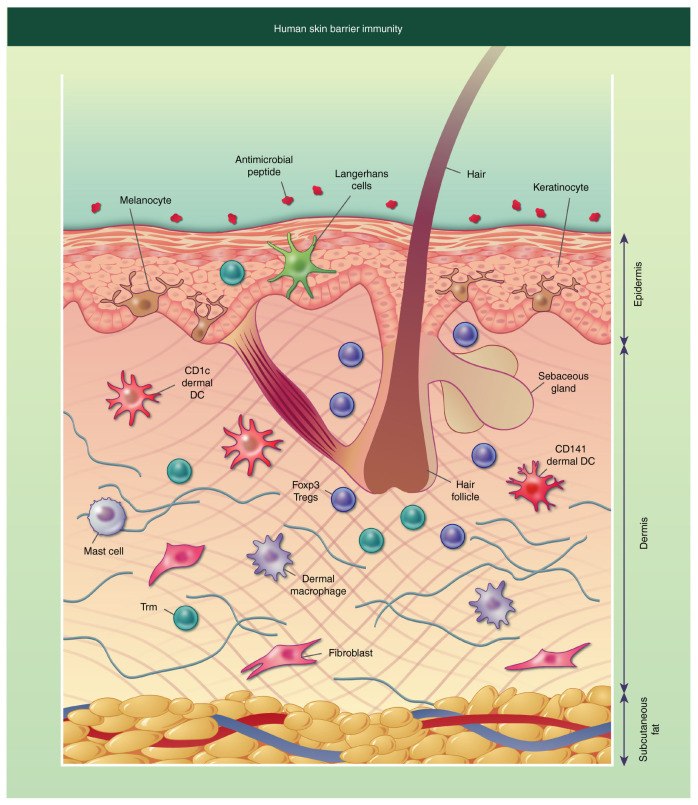
Schematic representation of human skin barrier immunity. The epidermal layer consists of T-resident memory cells (T_rm_), Langerhans cells, and keratinocytes; the latter form a stratified corneum with interspersed melanocytes. The dermal layer is populated by dermal dendritic cells (DCs), macrophages, Foxp3^+^ T regulatory cells (Tregs), CD4+ and CD8+ T_rm_, fibroblasts, and mast cells. The subcutaneous layer is composed of adipocytes. Illustration from Chambers et al., 2020 [23]. (License number 5835261420374).

Many immune cells, which are present in skin layers, work to respond to insults and injuries, contributing to cutaneous barrier immunity functions, as shown in Figure 14 [23].

**Figure 15:**
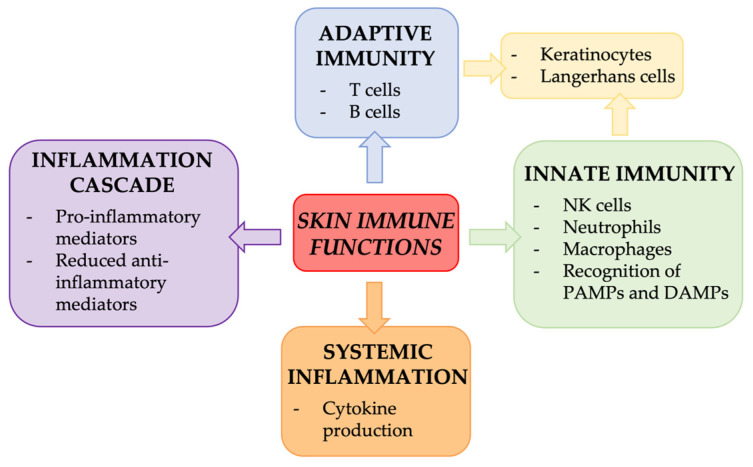
Skin immune functions. The graph summarizes the implications of immune cells for skin immune functions. Illustration adapted from Agrawal et al., 2023 [105].

They consist of various cell types, including lymphocytes, macrophages, granulocytes, mast cells, and dendritic cells [101]. Memory T cells, abundant in the epidermal layer, are lymphocytes that play a role in the early immune response, while circulating T cells, mostly present in the dermal layer, act against pathogens and participate in inflammatory processes [102]. Natural killers (NK) are cytotoxic lymphocytes able to kill tumor cells or cells infected with a virus. Macrophages, one of the most consistent types of skin’s immune cells, are specialized in the phagocytosis of pathogens and can have both anti-inflammatory and pro-inflammatory functions [103]. Neutrophils, as one of the varieties of granulocytes, fight bacterial infections and some fungal infections. Mast cells, after being activated by allergens, release inflammatory mediators, causing reactions such as itching and swelling. Dendritic cells play an important role in both the innate and adaptive immune responses, acting as antigen-presenting cells [101]. All the immune cells above reported work together to coordinate the cutaneous immune response, as shown in Figure 15 [104,105].

While the immune system ages, the numbers and functions of immune cells change, compromising the skin’s defensive functions. Macrophages and neutrophils undergo a reduction in their phagocytic and chemotactic functions, and this impairs their ability to regulate inflammation and microbial response. T cell functions also decline during aging, resulting in reduced cytotoxic action on aging cells. Moreover, the decreased ability of Langerhans cells to migrate reduces the detection of pathogens [101].

However, evidence of the aging of these cells during chronological aging are limited [31].

Recent research has identified that age-related decline in the functions of various skin immune cells is associated with inflammation, which is related to the removal of aging cells [106,107,108,109,110,111,112].

## 5. Pathomechanisms of Skin Aging

There is a symbiotic relationship between skin biology and metabolism; understanding this connection is a key element in unraveling the mystery of skin aging.

In this regard, the metabolic regulation of proteins, glucose, lipids, and iron levels can all impact skin aging processes [113]. For example, lipids are important components of all cell types, and they protect the epidermal structure of the skin against the external environment [114]. UV radiation may affect skin lipid homeostasis by inducing lipid peroxidation, brown pigment formation, and, thus, skin aging [115,116]. As a result, it is known that the appearance of the skin can affect the quality of life, both emotionally and psychologically, indicating that time passes [34].

### 5.1. Protein Metabolism

The synthesis of proteins and amino acids and their catabolism are important steps to maintain skin homeostasis [5,117].

As mentioned above, one of the first proteins involved in skin metabolism is collagen, which is responsible for maintaining its homeostasis [51,118].

As for amino acids, glutamine serves as a donor of energy and a nitrogen source required for purine/pyrimidine formation, and it is important for mitochondrial metabolism and cell regulation [20]. During starvation stress, glucose utilization is hindered, and proteins are degraded into glutamine or become an important source of energy for skin tissue [119]. Degradation of glutamine produces a replenishment substrate, but this substrate decreases with age, resulting in a slower metabolism of glutamine, which may affect the function and cytoarchitecture of the skin [114].

### 5.2. Glucose Metabolism

Glucose is an important fuel for skin energy production; it is used both for anaerobic and aerobic glycolysis [114,119]. Moreover, glycogen is synthesized from glucose and plays an energy storage role in the skin. When glucose is lacking in energy, the source of skin energy shifts to other fuels such as glycogen and lipids [5,119]. In aging skin, glucose metabolism slows down, resulting in a decrease in overall metabolic efficiency. This mechanism leads to increased AGE production, accelerating the process of skin aging [120].

### 5.3. Lipid Metabolism

Lipids are important components of all cell types, and they have several functions, such as protection, mitochondrial and cell membrane regulation, energy storage, and cell transduction [121,122]. In addition, fatty acids are very important components not only in lipid metabolism but also in the cytoarchitecture of skin metabolism, as reported by Shin et al. (2020) [114]. In aging skin, there is a slowdown in lipid metabolism and a reduction in protection of cutaneous structure. This process results in the formation of lipid peroxidation products, inducing aging processes and skin water loss [123,124].

### 5.4. Iron Metabolism

**Figure 16:**
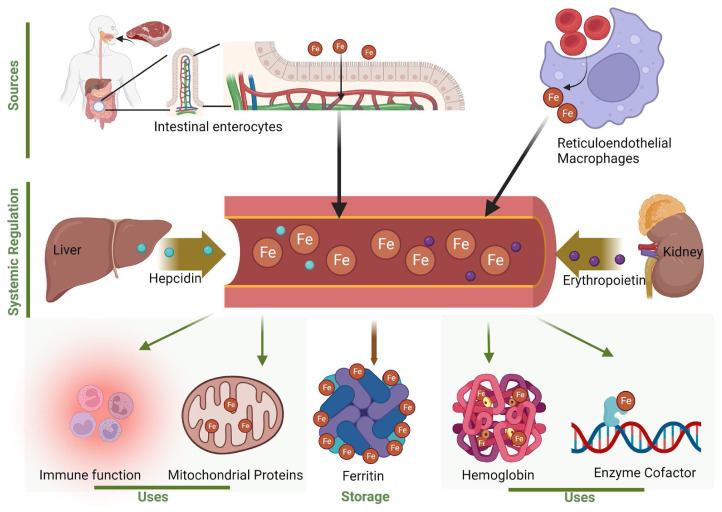
Iron homeostasis. The illustration represents an overview of the iron sources, the systemic processes that balance the iron level, and its cellular uses. The regulation of iron homeostasis involves the absorption, transport, storage, recycling, and utilization of iron. Illustration from Zeidan et al., 2024 [125]. (This is an open access article distributed under the terms of the Creative Commons CC-BY license).

Iron is a fundamental nutritional element; it is fundamental for several physiological functions [125]. Iron homeostasis is maintained through a complex system of regulation that involves the absorption, transport, storage, and utilization of this element [125] (Figure 16).

While iron levels are important for the physiology of several organs, their increase with age determines several disorders. In particular, increased intestinal permeability, colloquially referred to as “leaky gut,” is observed in the older adult population and has the potential to contribute to a systemic elevation in the pathological non-transferrin-bound iron [126]. This could be aggravated by dysbiosis of the gut microbiota in older adults; an increased iron level induces oxidative stress, resulting in a vicious cycle [125].

Table 3 summarizes the pathways and iron-related disorders in various organ systems. Iron overload and iron deficiency have distinct effects on organ systems, leading in some cases to oxidative stress, inflammation, and fibrosis [125].

**Figure 17:**
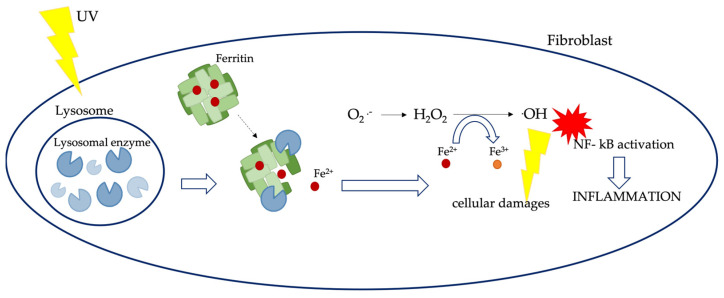
Ultraviolet irradiation and ferritin. The illustration represents the ultraviolet irradiation injury at skin level. The skin’s ultraviolet irradiation increases cell-free iron, promoting ferritin degradation, which, in a vicious cycle, implements the free iron release. (NF-kB): nuclear factor kappa-light-chain-enhancer of activated B cells. Illustration adapted from Pouillot et al., 2014 [127].

In addition, available free iron increases after UV irradiation in the skin through the degradation of ferritin molecules [127]. This release contributes to the direct toxicity of ROS; during UV-mediated oxidative stress damage, lysosomal membranes can be destabilized by lipid peroxidation, leading to lysosomal rupture and the release of hydrolytic enzymes into the cytosol. These enzymes damage ferritin, which releases free iron again (Figure 17).

## 6. Molecular Mechanisms in Skin Aging

As stated above, several mechanisms have been proposed to explain the biological pathways of skin aging, including the theory of cellular senescence, mitochondrial mutations, oxidative stress, inflammation, and a new RCD called ferroptosis.

Ferroptosis is involved in skin aging, but its mechanisms are not yet fully known. Therefore, we decided to emphasize its pathway and its involvement in skin aging without reporting the already-studied RCD. We added only a few sentences, hoping to specify their different pathways with respect to ferroptosis.

### 6.1. Cell Death for Skin Aging

Apoptosis and necrosis have been well studied in the skin [128]. Apoptosis does not account for the inflammation because it has anti-inflammatory and immunosuppressive effects [129]. It maintains cell membrane integrity, and it is caspase-dependent [130]. Necrosis, unsuch as apoptosis, induces the release of immunogenic proteins, triggering inflammation [128]. In the skin, other types of necrosis are pyroptosis and necroptosis; pyroptosis is a biochemical pathway inducing caspase-1–11 activation, whereas the necroptosis pathway activates receptor-interacting protein kinase 3 (RIPK3), which phosphorylates mixed-lineage kinase domain-like protein (MLKL) [131]. These mechanisms are characterized by membrane disruption [130].

Ferroptosis, first reported in 2012 as an RCD pathway, is induced by cellular changes in redox metabolism, iron handling, mitochondrial activity, and lipid metabolism, as well as numerous disease-relevant signaling pathways [12,13,14,130,132,133,134].

**Figure 18:**
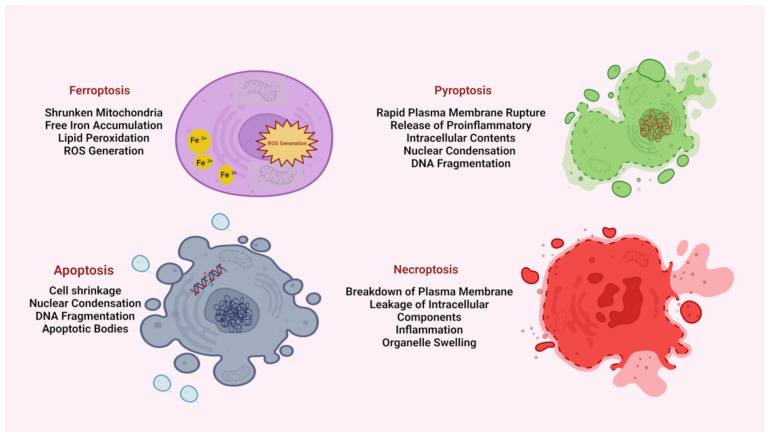
Programmed or non-programmed cell death. The illustration compares the main morphologic features of programmed and non-programmed cell death: ferroptosis, pyroptosis, apoptosis, and necrosis. Illustration from Khorsandi et al., 2023 [130]. (This is an open access article distributed under the terms of the Creative Commons Attribution 4.0 International license).

The main features of all these pathways are shown in Figure 18 [130].

#### 6.1.1. Ferroptosis as a New Cell Death Discover

**Figure 19:**
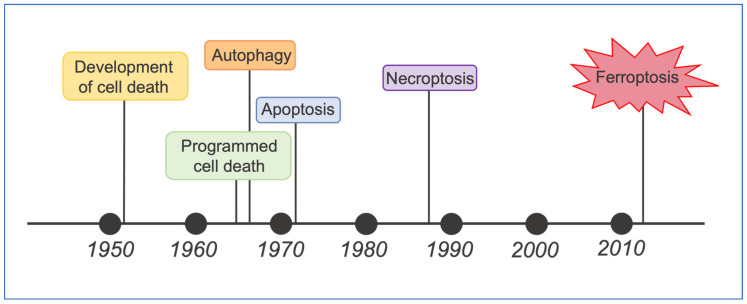
The development of the ferroptosis concept. Timeline diagram of last decades’ history observations on regulated cell deaths and the advances that contributed to the emergence of the concept of ferroptosis. Ferroptosis was observed in various contexts before coining of the term in 2012. Illustration adapted from Hirschhorn and Stockwell, 2019 [135].

Ferroptosis was discovered during a screening of lethal small-molecule compounds for oncogene evaluation [135,136,137]. It has been observed several times over the years before the detailed molecular understanding of this cell death process and the concept of its existence (Figure 19).

**Figure 20:**
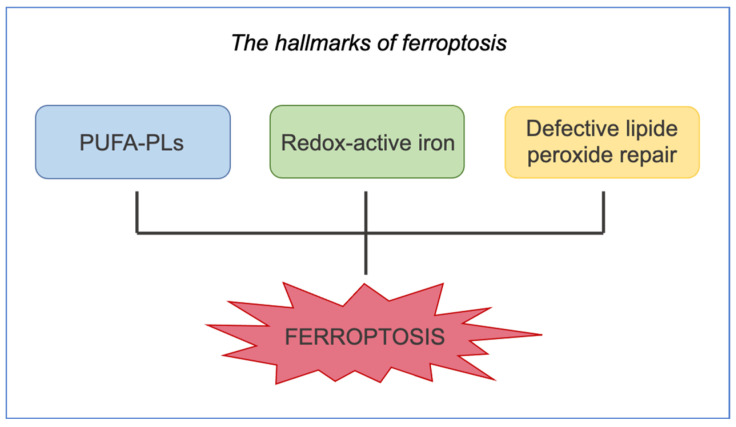
Ferroptosis hallmarks. The illustration summarizes the three main hallmarks that promote ferroptotic death: oxidizable phospholipids acylated with polyunsaturated fatty acids, redox-active iron, and defective lipid peroxide repair. Illustration adapted from Dixon and Stockwell, 2019 [143].

Ferroptosis has a distinctive morphological alteration compared with other RCDs; it shows smaller mitochondria, reduced inner mitochondrial membrane folds, increased membrane density of mitochondria, and loss of their cytoarchitecture [12,138]. Cells undergoing ferroptosis have normal nuclear size without chromatin condensation, nuclear fragmentation, or apoptotic bodies [139,140]. Iron, lipids, and ROS are important for maintaining a steady state for survival [141,142]. ROS accumulation causes lipid peroxidation, which induces ferroptosis. These distinctive morphological changes in ferroptosis help to distinguish it from other cell death pathways; Dixon and Stockwell (2019) suggested that there are three hallmarks of ferroptosis (Figure 20).

**Figure 21:**
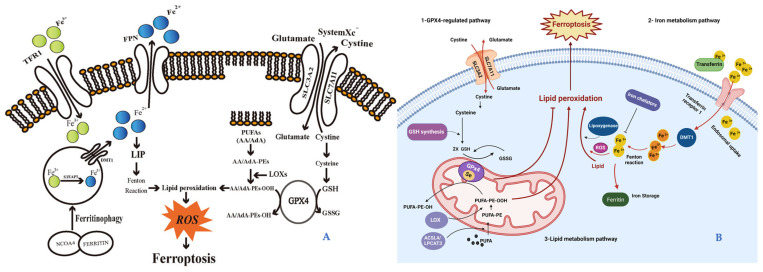
Ferroptosis cell pathways. The illustrations summarize the main conditions that can trigger cell ferroptosis. (TFR1): transferrin receptor 1; (FPN): ferroportin; (DMT1): divalent metal transporter 1; (STEAP3): six-transmembrane epithelial antigens of the prostate 3; (NCOA4): Nuclear receptor coactivator 4; (LIP): labile iron pool; (ROS): reactive oxygen species; (SLC3A2): Solute Carrier Family 3 Member 2; (SLC7A11): Solute Carrier Family 7 Member 11; (PUFAs): polyunsaturated fatty acids; (AA/AdA): arachidonic acid/adrenic acid (AdA); (AA/AdA-Pes): AA/AdAphosphatidylethanolamine (PE); (AA/AdA-PEs-OH): AA/AdA-PEs-alcohols; (AA/AdA-PEs-OOH): AA/AdA-PEs-hydroperoxides; (GPX4): glutathione peroxidase-4; (GSH): glutathione; (GSSG): glutathione disulfide; (LOXs): lipoxygenases; (ACSL4/LPCAT3): acyl-CoA synthetase long-chain family member 4/lysophosphatidylcholine acyltransferase 3. Illustration (**A**) is from Liu et al., 2023 [140]. (This is an open access article distributed under the term of the Creative Commons CC-BY license). Illustration (**B**) is from Khorsandi et al., 2023 [130]. (This is an open access article distributed under the terms of the Creative Commons Attribution 4.0 International license).

Recent research has illustrated that cells undergo ferroptosis, followed by changes in metabolism and regulatory mechanisms. Figure 21 shows a summary of the critical mechanisms and some regulators associated with ferroptosis in various aspects, including iron, system Xc-/glutathione, and lipid peroxidation regulation.

Ferroptosis is mainly stimulated by intracellular glutathione depletion and a decrease in the activation of glutathione peroxidase 4 (GPX4). Then, lipid peroxides are not metabolized by the GPX4-catalyzed reduction mechanism, and this results in an accumulation of lipid peroxides. Moreover, Fe^2+^ stimulates ferroptosis by oxidizing lipids, producing a large amount of ROS [144]. Furthermore, multiple genes have been identified to control ferroptosis, including the genes that control the prostaglandin-endoperoxide synthase (PTGS2/COX2), such as microRNA212 (MIR212)-mediated down-regulation of PTGS2 mRNA [137,138,145]. MIR212-mediated down-regulation of PTGS2 mRNA prevents ferroptosis neuronal death in a traumatic brain injury mouse model, as reported by Xiao et al., 2019. In fact, the authors demonstrated that, after treatment with specific inducers of ferroptosis (e.g., erastin), Ptgs2 is up-regulated, suggesting its cell type-dependent role in ferroptosis. Moreover, they showed that miR-212-5p suppresses ferroptosis in specific cell lines by targeting Ptgs2, among others.

A specific biomarker for ferroptosis is the enzyme acyl-CoA synthetase long-chain family member (ACSL4), which is involved in fatty acid family members. When ACSL4 is overexpressed, it triggers the oxidative stress-inducing ferroptosis pathway [146]. However, as depicted in Figure 18, this is not the rule due to various specific conditions that stimulate the cell to move toward the ferroptosis mechanism even when ACSL4 expression is low [130]. In Table 4, we collect the proteins involved in ferroptosis and their intracellular functions. In Figure 21A,B are schematically summarized the main conditions which can trigger to cell ferroptosis.

**Figure 22:**
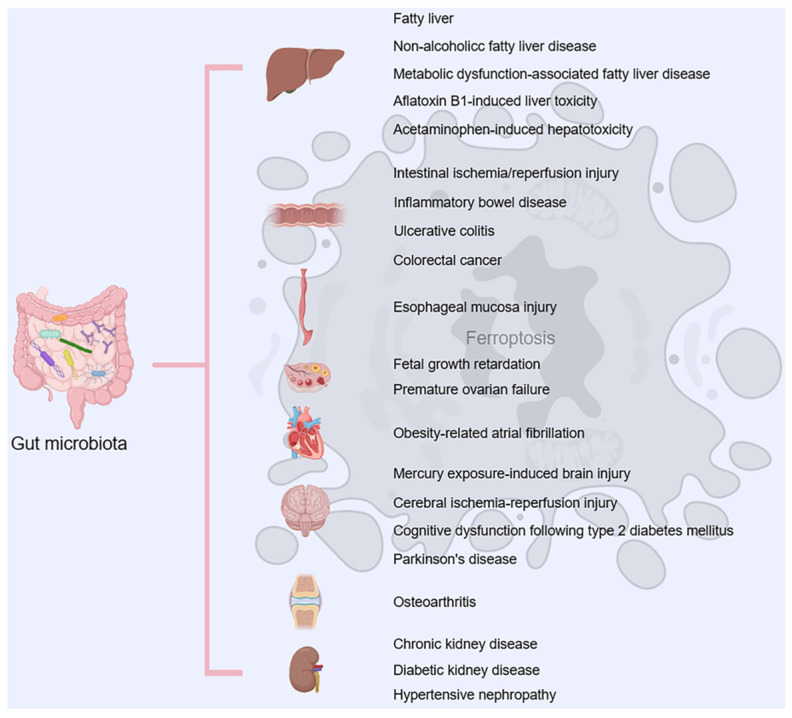
Ferroptosis and gut microbiota. The illustration represents the influence of gut microbiota on ferroptosis in various tissues, organs, and diseases. Illustration from Mao et al., 2024 [152]. (This is an open access article distributed under the terms of the Creative Commons Attribution 4.0 International license).

Ferroptosis is implicated in pulmonary, gastrointestinal, inflammatory, neurological, and circulatory diseases [147,148,149,150] and in hepatic alterations in autistic mice [151]. It has a strict link with the gut microbiota as reported by Mao et al. (2024) [152] (Figure 22). These authors suggested that the gut microbiota, has a significant impact on ferroptosis in various organs, such as the liver and intestine. Alterations in gut microbiota impact host metabolic homeostasis and the antioxidant system, suggesting that gut microbiota and its metabolites regulate ferroptosis. Exploring the effects of interventions that control gut microbiota, such as probiotics, prebiotics, and fecal microbiota transplantation, on ferroptosis, as well as new therapeutic approaches, is important for ferroptosis-related diseases [152].

To date, there are few papers reporting the involvement of ferroptosis in skin diseases. In the following paragraphs, we report current knowledge on the ferroptosis pathway in skin aging and pathology to better highlight its relevance. Knowledge of this cell death pathway could illuminate its role in the aging, pathogenesis, and treatments of these diseases.

#### 6.1.2. Ferroptosis in Skin Aging and Diseases

Papers reporting the involvement of ferroptosis in skin diseases and considering UV radiations promoting tumorigenesis, metastatic melanoma, and other skin pathologies are increasing [130,133,153,154,155]. In particular, researchers studied ferroptosis in melanoma, psoriasis, vitiligo, sclerosis diseases, and systemic sclerosis [14,133,140,148,156,157,158,159,160].

##### Ferroptosis and Melanoma

It is already known that UV radiation, as well as pesticides, herbicides, various aspects of lifestyle, and circadian rhythm disruption, can increase the risk of developing melanoma, which is the most aggressive form of skin cancer [155,157,161,162,163]. Melanoma cells show a highly plastic phenotype and a well-known capacity to differentiate into diverse tissue types [157,164]. In 2018, Tsoi et al. suggested that differentiation confers susceptibility to anticancer therapies despite promoting resistance to standard interventions [165]. These authors studied several melanoma cell lines, and they found that dedifferentiation status was correlated with sensitivity to ferroptosis inducers such as erastin. Finally, they demonstrated that proinflammatory cytokines such as tumor necrosis factor-α (TNF-α) and interferon-γ (IFN-γ) induce dedifferentiation and lethality following treatment with ferroptosis inducers. These results indicated that ferroptosis inducers promote antitumor immunity by killing dedifferentiated melanoma cells and preventing their immunosuppressive actions. It is unknown how the dedifferentiation leads to increased sensitivity to ferroptosis, but it is possible to speculate that TNF-α and IFN-γ could elicit down-regulation of system Xc- or glutathione synthesis enzymes or an unrelated pathway that modifies sensitivity to ferroptosis [157].

Vats et al. (2021) showed that keratinocytes have a very high susceptibility to UVB radiation in relation to dysregulation of the glutathione system [133]. The authors demonstrated that the inhibition of ferroptosis prevents the release of inflammatory proteins such as high mobility group box 1 (HMGB1) from human epidermal keratinocytes. HMGB1 is detected in the nucleus of skin epidermal cells and accumulates in the extracellular space during injury [166]. In the nucleus, HMGB1, as a non-histone nuclear protein, is involved in the regulation of several gene transcriptions [167]. When HMGB1 is released in the extracellular space, it acts as a cytokine by binding to the receptor for AGE (RAGE) and initiating a positive feedback autocrine circuit that maintains the inflammation processes [166]. These findings allow us to demonstrate that the inhibition of HMGB1 blocks inflammation in the UVB-irradiated mouse skin, suggesting that *ferroptosis* has important implications for the prevention and treatment of skin diseases due to UVB-induced inflammation [133].

Other melanoma considerations pointed to pheomelanin, a pigment produced by melanocytes in the epidermis, as the main culprit of UVA-induced ROS compared with another pigment such as eumelanin. This pigment mainly induces DNA damage, and thus, melanoma formation [168]. In recent decades, the lack of definitive treatments for this disease has required new therapeutic approaches for enhancing the survival rate of melanoma patients. Moreover, some patients benefit from treatment, while others do not respond to it. The development of personalized treatments would lead to improved patient survival and a low cost of patient care [130]. In this context, therapeutic approaches using ferroptosis still face challenges in guaranteeing an adequate survival fate for patients affected by melanoma [155]. Recently, it has been demonstrated that controlling ferroptosis may positively affect cancer treatment and overcome resistance [169,170]. Khorsandi et al. (2023) provided a complementary explanation of how ferroptosis can be accounted for and control drug resistance in melanoma [130]. Ferroptosis can start glutamate-induced cytotoxicity; therefore, iron chelators and other ferroptosis inhibitors can suppress glutamate-induced cytotoxicity in various ways. For example, glutamine is absorbed and converted into glutamate and α-ketoglutarate by several genes; their inactivation causes resistance to ferroptosis, and a decrease in glutamine synthesis and its accumulation in melanoma [171,172]. This mechanism induces anti-ferroptosis action on melanoma, as reported by Zhang et al. (2018) [173].

According to Sato et al.’s study, ferroptosis induces a deficiency of the cysteine-glutamate antiporter in melanoma cells. Deficiency of this system determines a reduction in cysteine uptake, cellular GSH, and subcutaneous tumor formation in vivo [172]. The ferroptosis inhibitor, liproxstatin-1, is not known to reverse these alterations. The authors suggested that melanoma metastasizing through the blood rather than the lymphatic system is dependent on GPX4 ferroptosis inhibitors [172].

##### Ferroptosis and Autoimmune Diseases

Several studies have considered the role of ferroptosis in psoriasis vulgaris (PV), which is a chronic and systemic skin disease characterized by erythematous plaques covered with silvery scales [174,175,176,177]. Shou et al. (2021) suggested that psoriatic keratinocytes can resist apoptosis, but they are more susceptible to ferroptosis [176]. As reported above, ferroptosis is mediated by lipid peroxidation, iron overload, and decreased levels of GPX4. Why GPX4 expression decreased in PV was unclear, but a possible explanation has been suggested for selenium depletion in these patients [178]. In addition to selenium deficiency, decreased levels of target rapamycin (mTOR1 complex) proteins have been found, and this inhibition sensitizes cells to ferroptosis. Therefore, the activation of this complex has been proposed as a possible positive mechanism to block the ferroptosis pathway [179]. Moreover, the same authors suggested that ferrostatin-1 (Fer-1), a potent inhibitor of lipid peroxidation, suppressed ferroptosis-related changes in PV, dermatitis that have been induced in mice. Zhou et al. (2022) reported an intricate relationship between ferroptosis and inflammation in PV suggesting that it not only promotes cell death but also triggers inflammation in psoriatic keratinocytes. The authors suggested considering this pathway to benefit psoriasis [177].

Some experimental reports have shown that epidermal GPX4 deletion induces increased lipid peroxidation and cyclooxygenase-2 levels in the whole skin, resulting in dermal inflammatory infiltration and epidermal hyperplasia in perinatal mice [160,180]. In this study, it was shown that ACSL4 was significantly increased in psoriatic lesions, and its expression was further up-regulated during disease progression. ACLS4 expression was directly correlated with the Psoriasis Area Severity Index (PASI) score and inflammation factors, such as TNF-α, interleulin-6 (IL-6), interleukin-8 (IL-8), and interleukin-17a (IL-17a). In addition, this marker promoted arachidonic acid, thereby increasing fatty acid peroxidation metabolites in cells and stimulating ferroptosis [181]. The abnormal increase of ACLS4 suggested that ferroptosis is a very important pathway in the progression of PV by amplifying the inflammatory state.

Other studies have shown that the dysfunction of epidermal keratinocytes plays an important role in PV etiology, although its involvement is not well studied [182]. These authors, using biochemical and ultrastructural analyses, suggested that abnormal expression of some genes related to iron metabolism results in the accumulation of ferrous ions in human epidermal keratinocyte (HaCaT) cells with the induction of ferroptosis. Ferrous ion overload and the ferroptosis pathway are responsible for skin lesions in people with PV. In conclusion, this study provided new clues to investigate the etiology of PV, although the underlying mechanisms need to be better investigated [182].

**Figure 23:**
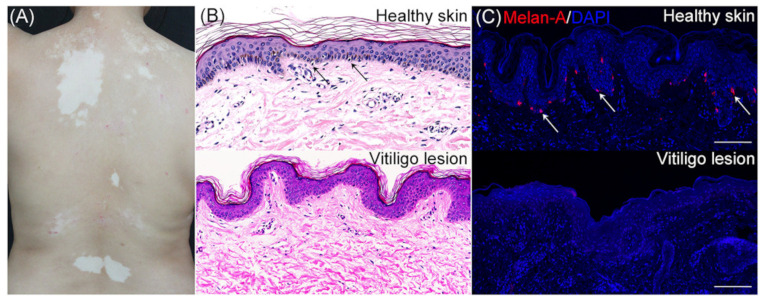
Dermatological manifestation of vitiligo. The illustration showed the main dermatological features of vitiligo. Cutaneous chronic depigmentation and white lesions of a vitiligo patient (**A**). Comparison of skin morphology between a healthy subject and a vitiligo patient. Haematoxylin-eosin staining. Black arrows indicate melanocytes (×400) (**B**). Comparison of skin melanocytes between a healthy subject and a vitiligo patient underling. Melan-A (red staining) immunofluorescence. Nuclei have been counterstained with 4′,6′-diamidino-2-phenylindole (blue staining). Bar = 100 µm. The white arrows indicated melanocytes (**C**). (**B**,**C**) underline the absence of melanocytes in vitiligo skin. Illustration from Chen et al., 2020 [158]. (This is an open access article distributed under the terms of the Creative Commons CC-BY license).

Vitiligo is an autoimmune disease of skin depigmentation with extensive melanocyte destruction [158,183] (Figure 23A). Moreover, the pathologic destruction of melanocytes is the main starting point of vitiligo, as shown in Figure 23B,C, and the way to modify this process has already been and is an important goal of basic vitiligo research [158].

**Figure 24:**
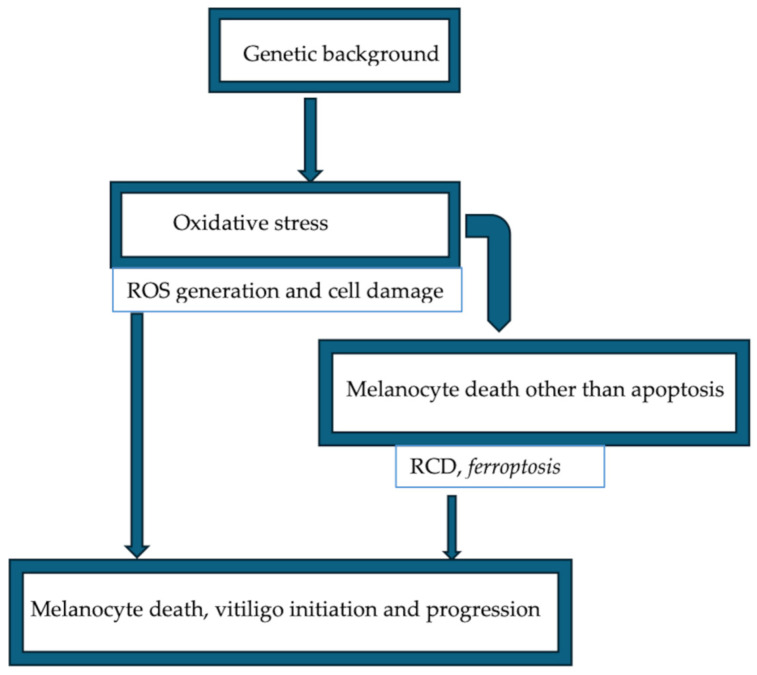
Melanocytes in vitiligo. The illustration represents schematically the main hallmarks that promote melanocyte destruction, resulting in the depletion of melanocytes in vitiligo lesional skin. (ROS): reactive oxygen species; (RCD): regulated cell death. Illustration adapted from Chen et al., 2020 [158].

In this context, investigations into the pathway of vitiligo death, likely to involve ferroptosis, necroptosis, and pyroptosis, will be very important to discover new therapies for vitiligo [158]. It is shown that a group of factors, such as GPX4 inactivation, p53 increase, ROS accumulation, innate and adaptive activation, and membrane lipid peroxidation in ferroptosis, contribute to melanocyte death [158,184] (Figure 24).

In addition, ferroptosis participates in IFN-γ-related cell destruction, and IFN-γ leads to melanocyte death, which has been shown to be an important hallmark in the etiopathogenesis of vitiligo [185,186]. These authors suggested that they were unsure whether ferroptosis directly contributes to melanocyte destruction in this disease. Other studies have stressed the importance of knowing the etiopathogenesis of vitiligo and how ferroptosis determines or can induce melanocyte destruction [187].

**Figure 25:**
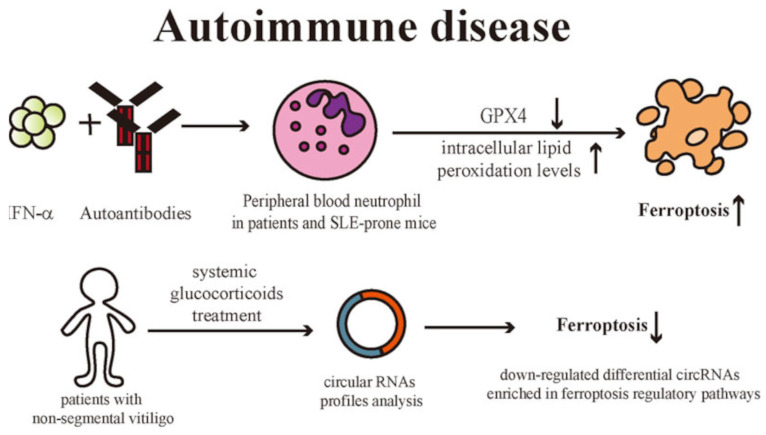
Autoimmune diseases and ferroptosis. The illustration summarizes the ferroptosis pathway in vitiligo-affected patients. (IFN-α): interferon-α; (GPX4): glutathione peroxidase-4; (SLE): systemic lupus erythematosus; (circRNA): circular RNAs. Illustration from Liu et al., 2023 [140]. (This is an open access article distributed under the terms of the Creative Commons CC-BY license).

Zhang et al. (2022) suggested that oxidative stress is one of the triggering factors for non-segmental vitiligo disease [188]. The authors studied the circular RNA (circRNA) expression profiles of glucocorticoid-treated and untreated non-segmental vitiligo patients. The profiles were significantly different, and the down-regulation of circRNA was predominantly enriched in the ferroptosis regulatory pathway. Therefore, they suggested that the ferroptosis pathway was inhibited in vitiligo patients and their results provided novel insights for the study of vitiligo, as shown in Figure 25 [140].

Systemic sclerosis (SSC) is a chronic autoimmune disease characterized by skin fibrosis, an important feature that leads to skin firmness and thickening [189]. The initial triggers of this disease are unclear, although the progression of skin fibroblasts and the production of matrix type I collagen are the main features of this disease [190,191]. Emerging pharmacological approaches are limited to targeting the organs that fall under this pathology [192,193,194]. For the first time, Zhang et al. (2024) demonstrated that GPX4 increased in SSC skin fibroblasts. Proliferation of these cells requires large amounts of iron, and the upregulation of GPX4 contributes to ferroptosis resistance. The authors suggested that GPX4 inhibition increases the sensitivity of SSC fibroblasts to ferroptosis, and then targeting ferroptosis is a therapeutic strategy for the treatment of SSC [194].

Systemic lupus erythematosus (SLE) is another autoimmune disease with several systemic symptoms [195,196]. One of the most often affected target organs is the skin; the most common skin signs are erythema of varying severity, irritation, or severe facial edema. SLE is characterized by the presence of immune complexes (IE) that induce inflammation and pathology in multiple organs [197,198]. The high number of IE affects the renal vessels, giving rise to lupus nephritis (LN), which is the most severe end-organ complication of SLE and induces end-stage renal disease (ESKD) [199,200]. Recently, several new mechanisms have been identified, one of which assessed iron metabolism and ferroptosis [201,202,203]. Alterations in critical substrates of ferroptosis, iron ion availability, and increased ROS levels are three pillars of this RCD pathway and important features in SLE/LN [202,204,205,206]. Specifically, in SLE, the metabolic alterations reorganize CD4+ T and B lymphocytes and their functions; iron accumulation results in increased cytokine production; ROS induces several damages in different organelles and alterations in lipid peroxidation [207,208,209]. Several publications indicate that ferroptosis is not only an important therapeutic target in SLE/LN, but knowing why cells decide to carry out ferroptosis sheds light on cell biology. Therefore, the identification of novel ferroptosis inhibitors is an important challenge for therapeutic strategies [210].

In our opinion, it is important to introduce the fact that ferroptosis is also associated with skin fibrosis, which is not related to autoimmune diseases, such as keloids and radiation-induced fibrosis [211,212,213].

Radiation therapy represents a common therapeutic approach for many tumors, and radiation-induced fibrosis is a related side effect often observed. This long-term sequela of radiation therapy is accompanied by skin retraction and induration, pain, necrosis, ulceration, and restricted range of motion, significantly impacting patients quality of life and leading to severe cosmetic and functional impairment [214,215]. Recently, Berry et al. (2024) demonstrated, for the first time, in vivo the occurrence of ferroptosis in skin following ionizing radiation injury. Ferroptosis contributes to the development of radiation-induced fibrosis, and attenuation of this process leads to reduced skin injury [211].

Keloid can occur after dermal trauma, leading to the growth of exogenous protrusions that invade adjacent normal skin. It is characterized by itching, pain, joint contractures, and limited mobility, affecting skin appearance and impacting quality of life [216,217]. Recently, Yang et al. (2024) measured iron content and the expression of ferroptosis-related genes including Solute Carrier Family 7 Member 11 (SLC7A11, also known as xCT), GPX4, transferrin receptor (TFRC), and nuclear factor erythroid 2-related factor 2 (Nrf2) in keloid tissues, comparing the data to normal skin tissue.

Furthermore, the authors evaluated the role of ferroptosis in the development of keloid through the use of a ferroptosis inhibitor (ferrostatin-1) and a ferroptosis activator (erastin). Keloid tissues exhibited, respect for healthy skin, a high level of iron, and altered expression of SLC7A11, GPX4, Nrf2, and TFRC. These data indicate that ferroptosis is present in keloid and may be involved in its onset and progression.

Furthermore, ferrostatin-1 prevented fibrosis in keloid, inhibiting fibroblast ferroptosis, while erastin promoted fibrosis in keloid, intensifying ferroptosis in fibroblasts [212]. However, there are few studies on the role of ferroptosis in keloid, and further investigations are needed.

## 7. Concluding Remarks and Perspectives

**Figure 26:**
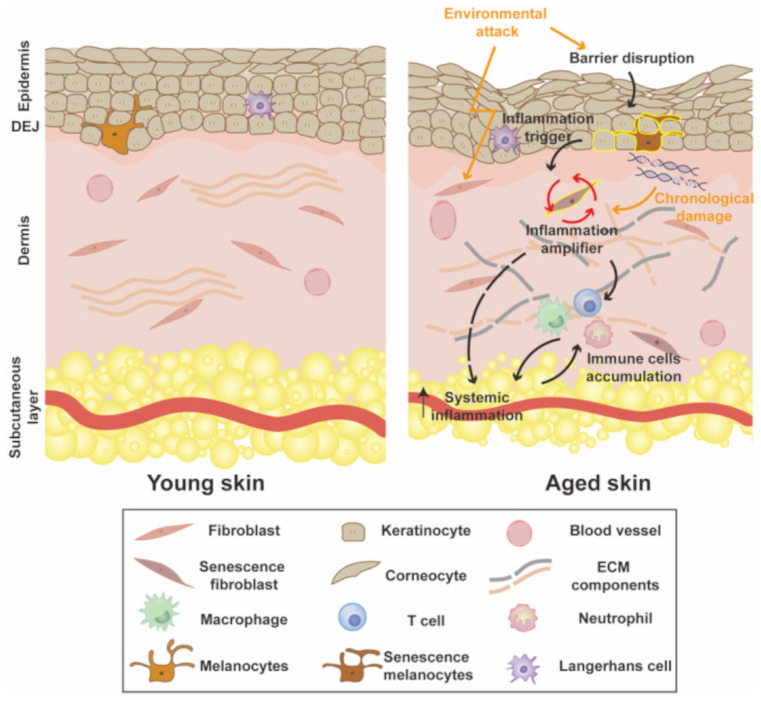
Young vs. old human skin. The illustration compares human young skin to human aged skin. Aged skin is characterized by an altered epithelial barrier, a thin and impaired dermal layer, inflammation, and excessive senescent fibroblasts and immune cells. Illustration from Zhang et al., 2024 [219]. (This is an open access article distributed under the terms of the Creative Commons CC-BY license).

The skin acts as a physical barrier that protects the body from external insults. Exposure of the skin to external insults induces significant changes throughout life, differentiating the skin of a child from that of an adult. These changes are caused by a combination of intrinsic aging, known as chronological aging, and extrinsic aging brought about by environmental factors such as air pollution, UV radiation, and other insults [194]. Senescence of epidermal keratinocytes and dermal fibroblasts determines or contributes to skin aging [35,124,218,219] (Figure 26).

In this regard, we highlighted the interaction between skin aging and its metabolism, showing that it is due to inflammation, ROS, AGE product accumulation, lipids, iron-dependent cell death, and ferroptosis [14]. The studies highlighted in this review suggest that the iron metabolism supports cellular survival, but when it is dysregulated, it can cause ferroptosis, contributing to skin pathology and more. We have emphasized and focused on dysregulation of iron metabolism in skin diseases (vitiligo, PV, melanoma, SSC, and SLE), arguing that this RCD is not well understood and that new technologies on its markers are important to design new treatments for iron overload and age-related diseases. Moreover, the challenge is to turn basic research into clinical applications by identifying the link between ferroptosis, senescence, aging, and disorders. Overall, the regulation of this pathway may give a deeper insight into the role of ferroptosis in skin diseases and help provide and optimize therapeutic options.

## Figures and Tables

**Table 1:** Content of type I and type III collagen in normal skin (mean ± s). The table summarizes type I and type III collagen content at the dermal layer, expressed as (mean ± s). The dermal collagen type varied significantly among the four age groups, and the ratio of type I to type III collagen increased with patient age. Different letters indicated statistically significant differences in mean across age groups (*p* < 0.05). Table adapted from Cheng et al., 2011 [51]. (This is an open access article distributed under the terms of the Creative Commons CC-BY license).

Age Group	N° Specimens	Type I Collagen (µg/g)	Type III Collagen (µg/g)	Type I/III
Fetus	10	264.71 ± 5.88 ^a^	278.87 ± 6.18 ^a^	0.95 ± 0.03 ^a^
Adolescent	10	279.12 ± 7.65 ^b^	123.27 ± 5.30 ^b^	2.27 ± 0.13 ^b^
Adult	10	241.79 ± 8.2 ^c^	98.41 ± 5.58 ^c^	2.46 ± 0.15 ^c^
Elderly	10	209.50 ± 14.31 ^d^	71.30 ± 7.41 ^d^	2.97 ± 0.40 ^d^

**Table 2:** Main anti-inflammatory compounds with anti-aging activity. Table adapted from Wollina et al., 2017 [92].

Source	Compounds
Aloe vera	Aloe sterols
Anemarrhena asphodeloides Bunge	Crude extracts and pure compounds containing steroidal saponins, flavonoids, phenylpropanoids, alkaloids, steroids, organic acids, anthraquinones and other compounds
Arctium lappa	Arctiin
Chlamydomonas hedleyi	Mycosporine-like amino acids
Glycyrrhiza glabra	18*β*-Glycyrrhetinic acid
Patchouli	Pogostone from patchouli oil

**Table 3:** Iron dyshomeostasis and its effects on various organs. Table adapted from Zeidan et al., 2024 [125].

Organ System	Mechanism	Associated Pathologies
Liver	Iron overload-induced hepatic fibrosis; iron deficiency reduces activity of cytochrome P450 enzymes involved in drug metabolism	Liver cirrhosis, hepatic-hypoxia
Kidneys	Excess iron accumulation causes iron-mediated oxidative stress and subsequent renal fibrosis; leading to impaired renal cell fraction	Chronic kidney disease
Brain	Excessive iron accumulation has been linked to increased risk of neurodegenerative disorders	Parkinson’s disease, Alzheimer’s disease
Heart	Iron is involved in oxygen transport and utilization; iron overload causes oxidative stress, inflammation and fibrosis	Atherosclerosis
Pancreas	Iron-induced oxidative stress and advanced glycation end products impair insulin signaling and β cell dysfunction with impaired insulin secretion	Type 2 diabetes mellitus
Joints	Iron is a component of several enzymes and proteins involded in cartilage and bone formation, maintenance and repair; iron deficiency induces alterations in cartilage and bone formation	Osteoarthritis, joint pain
Endocrine glands	Iron deficiency leads to decreased hormone production and secretion	Hypothyroidism, sexual dysfunction, infertility

**Table 4:** Proteins and their intracellular functions in ferroptosis.

Proteins and Regulators	Intracellular Functions Involved in Ferroptosis
Solute Carrier Family 3 Member 2 (SLC3A2) Solute Carrier Family 7 Member 11 (SLC7A11)	The constituents of an amino acid antiporter (Xc- system) that mediates the exchange across the plasma membrane of extracellular cystine and intracellular glutamate. Cystine is then reduced to cysteine and takes part in the synthesis of GSH, the substrate of GPX4;
Glutathione peroxidase 4 (GPX4)	A decrease in its activity causes lipid peroxides to be unable to be metabolized, resulting in lipid peroxides accumulation and inducing ferroptosis;
Acyl-CoA synthetase long-chain family member/ lysophosphatidylcholine acyltransferase 3 (ACSL4/LPCAT3)	ACSL4 catalyzes the ligation reaction of CoA with AdA/AA forming COA-AdA/AA. LPCAT3 catalyzes the esterification of COA-AdA/AA with lysophospholipids. Its overexpression triggers oxidative stress inducing ferroptosis;
Lipoxygenases (LOXs)	A group of iron-containing enzymes responsible for catalyzing the PUFAs oxidation through stereo-specific peroxidation to produce fatty acid hydroperoxides. Suppression or downregulation of LOXs activity leads to inhibition of ferroptosis in certain cell lines;
Transferrin receptor 1 (TRF1)	Mediates TF- Fe^3+^ transport through the membrane;
Ferroportin (FPN)	Iron efflux pump that can release intracellular iron by oxidizing it;
Divalent metal transporter 1 (DMT1)	Translocates Fe^2+^ into a labile iron pool (LIP) within the cytoplasm;
Six-transmembrane epithelial antigens of the prostate 3 (STEAP3)	Reduces Fe^3+^ in Fe^2+^ in the endosomes;
Nuclear receptor coactivator 4 (NCOA4)	Autophagic cargo receptor of ferritin;
